# Assessment of Biofilm Growth on Microplastics in Freshwaters Using a Passive Flow-Through System

**DOI:** 10.3390/toxics11120987

**Published:** 2023-12-05

**Authors:** Chengyang Jiang, Husein Almuhtaram, Michael J. McKie, Robert C. Andrews

**Affiliations:** Department of Civil and Mineral Engineering, University of Toronto, 35 St. George Street, Toronto, ON M5S 1A4, Canada

**Keywords:** biofilm, weathering, ATP, metagenomics, freshwater, PVC, PP, PET, HDPE, LDPE

## Abstract

Biofilms that colonize on the surface of microplastics (MPs) in freshwaters may pose a potential health risk. This study examined factors that influence MP-associated biofilm growth, including polymer type, degree of weathering, and source water quality. Weathered MPs produced in-lab were employed in biofilm trials conducted on site using a passive flow-through system with raw water at drinking water treatment facility intakes. Adenosine triphosphate (ATP) was used to quantify biofilm abundance; biofilm composition was assessed via metagenomic sequencing. Biofilm growth was observed on all polymer types examined and most prevalent on polyvinyl chloride (PVC), where ATP levels were 6 to 12 times higher when compared to other polymers. Pathogen-containing species including Salmonella enterica and Escherichia coli were present on all polymers with relative abundance up to 13.7%. *S. enterica* was selectively enriched on weathered MPs in specific water matrices. These findings support the need to research the potential accumulation of pathogenic organisms on microplastic surfaces.

## 1. Introduction

Until recently, the definition of microplastics (MPs) has been ambiguous with the exception of a commonly adopted “5 mm” upper size limit [1]. In 2020, the California Water Board defined MPs in drinking water as solid polymeric materials with at least three dimensions greater than 1 nm and less than 5 mm [2]. Consensus however exists that MPs are ubiquitous in the environment and have been observed with increasing frequency in freshwaters as well as treated drinking water, raising concerns regarding human exposure and potential associated health risks [3,4,5,6].

The World Health Organization (WHO) reported three types of potential MP-associated hazards: (i) physical hazard associated with polymer size, (ii) toxicity associated with polymer composition, and (iii) biofilms that may attach and colonize [7]. The focus of the current study is to examine factors that influence MP-associated biofilm. This topic is of concern due to the potential role of (MP-associated) biofilm as carriers of pathogens including *Vibrio*, *E. coli*, *Pseudomonas*, and *Arcobacter* [8], sinks for antibiotic resistant bacteria and genes [9], contributors to the sorptive capacity of chemicals in source waters [10], as well as creating physical barriers during drinking water disinfection [11,12]. Microplastics in marine environments have been shown to serve as surfaces that enable the growth of bacteria and potentially pathogenic organisms including *Vibrio* spp. [13,14].

To date, only a few of the MP-associated biofilm studies investigated freshwaters, most of which arose from Asia and Europe [15,16,17,18,19]. Meanwhile, although studies do exist in North America regarding MP biofilms, they focused mostly on wastewater [20,21,22,23]. This is of importance since biogeography can have a significant impact on biofilm formation [17,24]. Specific factors that impact biofilm formation on MPs in freshwaters are poorly understood, and existing studies have made little or no attempt to differentiate among specific MP polymer types. One study examined the impact of environmental conditions on MP biofilm assemblages; however, freshwater matrices were not considered [17].

Another important limitation is associated with the in situ sampling and analysis of environmental MPs. Most trials have been conducted at the bench scale using virgin MPs and natural or simulated water [14,15,16,25]. One study examined MPs of various sizes (<250 µm and 250–500 µm) and polymer types (polystyrene and polyethylene) in North American river water; however, trials were limited to the use of virgin MPs in 100 mL batch reactors [26]. As such, a need exists to assess biofilm growth in situ for both virgin and weathered MPs.

As it is challenging to collect appropriate environmental MPs that may be distinguished by polymer type, weathered MPs should be considered as an alternative. Weathering involves subjecting virgin MPs to abrasion and photooxidation to simulate environmental conditions. UV-weathered polyethylene has been reported to increase the induction of oxidative stress in cell-based bioassays when compared to control groups [27]. During an incubation trial that incorporated inoculated microcosms including *E. coli*, weathered polypropylene MPs were associated with higher bacterial abundance when compared to virgin polymers, which is likely attributed to changes in surface roughness and other physicochemical properties [28]. As such, a need exists to investigate biofilm formation using weathered microplastics [29].

The primary objective of the current study was to identify factors that impact MP-associated biofilm growth, including polymer type, degree of weathering, and source water quality. In situ trials were conducted using weathered and virgin MPs at four sites representing different source waters in southern Ontario, Canada. Weathered MPs were prepared from virgin MPs such that biofilms could be examined for both in terms of abundance and composition. The results of this study provide insight regarding biofilm growth on MPs in freshwaters.

## 2. Materials and Methods

### 2.1. Overview of In Situ Trials

A passive flow-through system (Figure S1a) was designed to allow biofilm development on various types of virgin and weathered MPs. During in situ trials, flow-through systems were installed on site and supplied a continuous flow rate of approximately 300 L/h using the influent of four water treatment plants associated with the following freshwaters: Lake Ontario, the Otonabee River, the Grand River (upstream) and the Grand River (downstream) (Figure S1b). In situ trials were conducted with exposure periods of up to 21 weeks. Historical raw water quality parameters pertaining to inorganic nutrients are provided in Table S1, and water temperature along with other water quality parameters for in situ trials are provided in Table S2.

High-density polyethylene (HDPE), low-density polyethylene (LDPE), polypropylene (PP), polyethylene terephthalate (PET), and polyvinyl chloride (PVC) were selected based on results from open-water surveys of plastic debris [30,31], reports of MP occurrence in drinking water [32,33], as well as the commercial availability of polymers [34,35]. Laboratory-grade microplastics were specifically avoided due to their lack of resemblance to environmental MPs; instead, generic pre-production polymer pellets (virgin MPs) used in the manufacturing of plastics were employed. All particles were within the size range of 3.0 to 3.5 mm (Table S3). Polymer composition was confirmed using Fourier-transform infrared spectroscopy (FTIR). Virgin microplastics were used as received without pre-treatment. Weathered MPs were produced in lab using these virgin MPs and were incorporated in biofilm trials.

### 2.2. Microplastic Weathering

Ideally, MPs that have undergone natural weathering (i.e., environmental MPs) should be employed; however, it would be impossible to obtain virgin polymers with the same properties for comparison. Other studies have utilized limited quantities of identifiable eroded LDPE and PP obtained from a beach but did not provide details regarding comparison virgin polymers used as controls [28]. As such, in the current study, weathered MPs were produced using an in-lab apparatus (Figure S2) adapted from a previous study that has demonstrated the efficacy to simulate weathering under natural conditions [36]. The system considered both hydrolytic and photooxidative weathering while avoiding the use of unrealistic exposure conditions, and it was calibrated to mimic weathering conditions in North American freshwater (Figure S3 and Table S4). Preliminary weathering trials were conducted for up to 24 weeks. Based on the results, a period of 6 to 8 weeks was selected to produce weathered polymers used in the biofilm trials.

### 2.3. Analyses of Weathered Polymers

Microscope images of virgin and weathered microplastics were obtained using a HORIBA XploRA PLUS Raman system (Kyoto, Japan) equipped with 10× and 50× working distance objectives (Olympus, Tokyo, Japan) and processed using LabSpec6 software. The surface roughness of microplastics was calculated as the mean surface deviation in the *z*-axis measured by a KLA Tencor P16A Stylus Profilometer (Milpitas, CA, USA). To avoid the interference by particle shape, a 500 nm linear section from the scan was selected and fit to a line that was used as the *z*-axis. Mean surface roughness (Table S5) was calculated as the average of deviation (in absolute value) from the *z*-axis at each point.

FTIR was used to characterize MP surface changes during weathering trials. Analyses were conducted using a Spectrum Two spectrometer with an attenuated total reflection (ATR) attachment (PerkinElmer, Waltham, MA, USA). FTIR spectra were obtained with a range of 4000–400 cm^−1^, a resolution of 1 cm^−1^, and 30 scans per individual particle. All spectra were background-, ATR-, and baseline-corrected as well as normalized to the maximum peak. To quantify spectral changes following weathering, bond indices were calculated by integrating the band area of a specific functional group, usually an oxidation product, normalized to a stable reference peak (formula provided in the Supporting Information). Indices of carbonyl (C=O), vinyl (C=C), hydroxyl (O−H), and carbon–oxygen (C−O) groups were calculated as described in the literature [34,36,37,38]. For cases where peaks associated with carbonyl and vinyl groups were inseparable, a combined C=X index was calculated [34,36].

X-ray photoelectron spectroscopy (XPS) analyses were performed to further characterize MP surface oxidation conditions using a K-Alpha XPS system (ThermoFisher Scientific, East Grinstead, UK) equipped with monochromatized Al K-Alpha X-ray source and a 300 µm spot size (2:1 ellipse). Data were acquired at a nominal take-off angle of 90 degrees. Survey spectra were first acquired at a pass energy of 200 eV (1 eV step size) to identify all species on the surface. Subsequently, regional scans were performed at 50 eV with a 0.1 eV step size for quantification purposes, and the dwell time for spectra acquisition was 50 ms. All data were processed using Thermo Scientific Avantage software. Surface elemental compositions were calculated from background-subtracted peak areas that were derived from transmission-function-corrected regional spectra. Sensitivity factors used to calculate the relative atomic percentages were provided by the instrument manufacturer.

Surface roughness results were obtained from ten randomly selected particles of each MP sample type; three replicates were used for all the other weathering analyses described above.

### 2.4. Biofilm Abundance

Adenosine triphosphate (ATP) represents a measure of cellular energy and has been used to characterize biological growth on microplastics [39]. ATP associated with biofilms attached to microplastics (1 g, or approximately 20 particles) was quantified in duplicate using a DSA-100C Deposit & Surface Analysis kit (LuminUltra, Fredericton, NB, Canada) following instructions provided by the manufacturer.

### 2.5. Biofilm Metagenomics

Metagenomic analyses were used to characterize biofilm composition due to its ability to provide detailed information at the species level. Analyses were conducted on a subset of polymers representing different exposure times and weathering conditions. For a given polymer type, DNA was extracted from 20 individual particles using a Qiagen DNeasy PowerBiofilm Kit (Hilden, Germany) following instructions provided by the manufacturer except for one deviation: 50 µL of solution “EB” was used to elute DNA from the filter membrane instead of 100 µL. Extracted DNA was stored at −80 °C prior to analysis.

Illumina libraries were prepared using an Illumina DNA kit (San Diego, CA, USA). Libraries were dual-barcoded with the Nextera^®^ XT Index kit and quantified using the Qubit HS DNA kit (ThermoFisher Scientific) pooled in equal amounts. The final pool was denatured and diluted to a sequencing concentration of 1.8 pM. Sequencing was performed on an Illumina NextSeq using the V2 chemistry with 150 × 2 PE reads.

Sequences were trimmed to remove adapters and low-quality sequences using Trimmomatic with default parameters, an average quality minimum of 20, and a minimum sequences length of 125 bp [40]. Cutadapt was used to remove stretches of sequences that contained homopolymers of G base. PCR duplicates were identified and removed using PrinSeq [41]. Unmapped, non-human reads were processed as the microbial metagenome. Kraken2 with default settings was used to assign taxonomic labels to the mapped sequences of each sample [42]. Bracken was then employed to estimate the species abundance of samples summarized at each taxonomic level [43].

The beta diversity of biofilm communities was compared using principal coordinate analysis (PCoA) based on Bray–Curtis dissimilarities using R [16]. Confidence ellipses were generated at 95% confidence using the ggplot2::stat_ellipse() function.

### 2.6. Water Quality Parameters

Dissolved organic carbon (DOC) was measured based on Standard Method 5310 D using an O-I Corporation Model 1010 Analytical TOC Analyzer (College Station, TX, USA) equipped with a Model 1051 Vial Multi-Sampler [44]. Ultraviolet absorbance at 254 nm (UV254) was measured using an Agilent 8453 UV–VIS spectrophotometer (Agilent Technologies, Mississauga, ON, Canada) with a 1 cm quartz cuvette (Hewlett Packard, Mississauga, ON, Canada). pH was measured using an Orion Star A111 Benchtop pH Meter (Thermo Scientific, Mississauga, ON, Canada). Turbidity was measured using a HACH 2100N Turbidimeter (Loveland, CO, USA). Temperature was recorded at 10-min intervals during in situ trials using an OM-EL-USB-TP-LCD temperature probe data logger (Omega Engineering, Stamford, CT, USA). All parameters mentioned above, except water temperatures, were measured in triplicate.

### 2.7. Statistical Analysis

Unless indicated otherwise, all comparisons between groups of samples were conducted by applying one-way repeated measured analysis of variance (ANOVA) and Tukey’s honest significant difference (HSD) post hoc tests at the 95% confidence level.

## 3. Results and Discussion

### 3.1. Characterization of Weathered Microplastics

Microscope images provided a visualization of physical weathering for all five polymer types (Figure S4). Using a 10× objective lens, cracks, chips, and scratch marks were observed on weathered HDPE, LDPE, PVC, and PP samples but to a lesser degree on PET, indicating possible increases in surface area. The difference in surface roughness between virgin and weathered microplastics was examined using a two-tailed *t*-test (Table S5). Among all the polymer types examined, roughness seemed to follow the order of PP > PET > PVC > LDPE ≈ HDPE. Weathering did not cause a significant impact (*p* < 0.05) on surface roughness except for PP, which increased in roughness following weathering. Changes in surface characteristics were attributed to a combination of physical abrasion associated with sand particles and polymer deterioration due to photo-induced oxidation and hydrolytic degradation [45].

Based on the FTIR results, weathered HDPE, LDPE, and PP shared similar patterns of bond index changes throughout the 24-week weathering period; all indices peaked following an initial 6 to 8 weeks (Figure S5). This was anticipated when considering the resemblance of their polymeric structures containing carbon–carbon backbones [45]. Weathered PVC showed increasing trends only for vinyl and hydroxyl indices with peaks appearing after 4 to 6 weeks. However, the carbonyl indices of weathered PVC polymers showed little to no variation during the same period. No apparent impact of weathering was observed when considering the bond indices of weathered PET, which generally showed a decreasing trend albeit with fluctuation. Although theoretical increases of these indices were anticipated, it is not uncommon to observe unchanged PET spectra following weathering attempts, as reported by others [46].

The oxygen/carbon (O/C) ratio was calculated as a quantitative measurement of weathering [47,48] based on atomic composition acquired via XPS analyses (Figure S6). All polymers showed increases in the O/C ratio following only 4 weeks of weathering. For HDPE, LDPE, and PET, the O/C ratios after 4, 8, and 16 weeks were consistently higher when compared to those of virgin polymers, illustrating the impact of oxidation. However, O/C ratios for PVC and PP decreased by varying degrees beyond 8 weeks.

Overall, signs of weathering (as a result of oxidation) following 6 to 8 weeks were evident for all MPs analyzed using FTIR and/or XPS. A cyclic weathering process was observed whereby virgin surface layers underwent oxidation and eventually deteriorated, exposing new virgin layers underneath (Figure S7). Weathering continued beyond 6–8 weeks for some polymer types; however, there were a greater number of changes when considering C=X, C-O, and O-H bond indices within this time period. Hence, 6 to 8 weeks was selected for the production of weathered microplastics for use in biofilm-related trials. Since the weathering method was adopted from a published study with detailed evidence of its efficacy [36], the current study observed similar results generated by FTIR as indicated by the reference study and thus confirmed the presence of polymer weathering.

### 3.2. Impact of Polymer Type on Biofilm Growth

**Figure 1:**
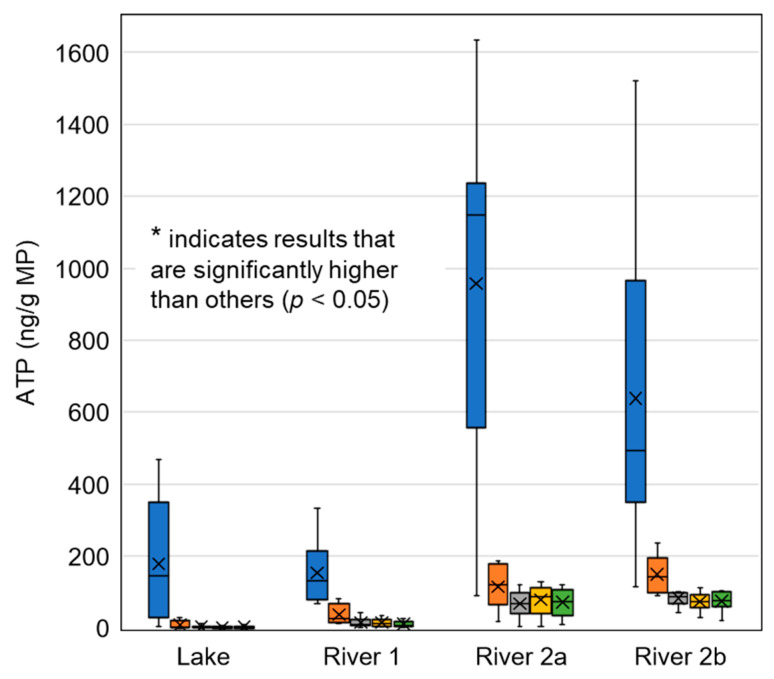
ATP associated with five virgin polymer types and four source waters. Vertical bars indicate maximum and minimum values, boxes indicate upper and lower quartiles, “×” indicates mean value, “−” indicates median. *n* = 6 for Otonabee River and *n* = 8 for all others.

Microplastics of all polymer types, regardless of weathering condition or water matrix, showed evident biofilm growth as indicated by ATP (Figure 1). Observed concentrations associated with PVC were significantly higher when compared to other polymer types (*p* < 0.05), averaging 178, 154, 958, and 640 ng/g among Lake Ontario, the Otonabee River, the Grand River (upstream), and the Grand River (downstream) (water quality parameters provided in Tables S1 and S2), respectively. In contrast, the average observed ATP levels for other polymers ranged between 42 ng/g (PP) and 80 ng/g (LDPE). Furthermore, LDPE results appeared to be higher than PET, HDPE, or PP, but this trend was not statistically significant (α = 0.05); and no differences were observed among PET, HDPE, and PP using one-way repeated measures ANOVA (α = 0.05). These results further suggest that PVC is more amenable to biofilm growth when compared to other polymer types.

A general consensus exists among studies with respect to impact of material surface properties on biofilm formation, with surface roughness and hydrophobicity serving as the two predominant parameters [17,49,50]. In theory, surfaces having higher roughness and hydrophobicity are likely to promote biofilm growth. Rougher surfaces provide additional surface area for biofilm attachment and reduce detachment by decreasing shear forces within the boundary layer [51,52]; hydrophobic microorganisms are more likely to attach to hydrophobic substrates in order to minimize contact with water [52,53]. However, actual biofilm growth patterns are not always predictable. For example, studies have reported “no difference” in biofilm formation when comparing PVC and HDPE pipe coupons in chlorinated distribution systems [54] as well as “no difference” in biofilm abundance on hydrophilic glossy steel surfaces and hydrophobic PVC surfaces [51].

A possible explanation is that the variation in surface properties is more impactful during the initial stages of biofilm development (up to 4 months) when early colonizers attach, but it becomes less important following extended periods as successive layers of biofilms begin to form [17,51,52,55]. In the current study, the polymer type with the highest ATP levels (PVC) was theoretically less hydrophobic than PE or PP [56]. As such, in this case, the impact of polymer type on biofilm formation could not be attributed to hydrophobicity, indicating a need to further characterize surface roughness as well as investigate other factors. These include polymer composition, coating, electrostatic properties, and the micropatterning of surfaces as well as the ability to form hydrogen bonds with microorganisms [52,53].

### 3.3. Impact of Water Matrix on Biofilm Growth

**Figure 2:**
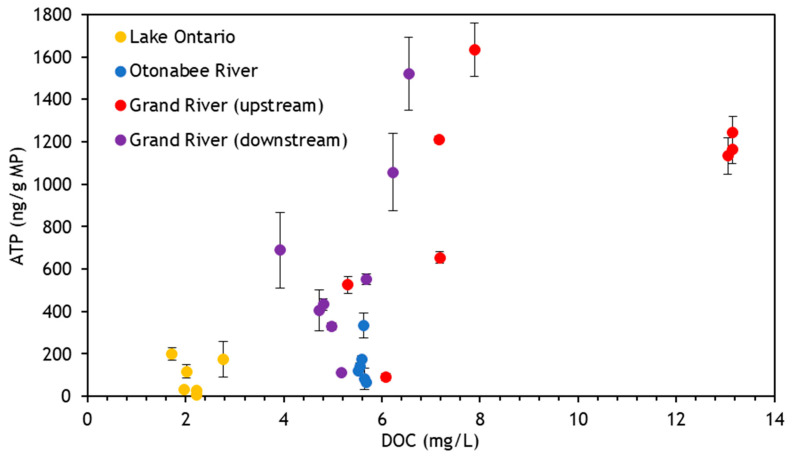
Correlation between (virgin) PVC-associated ATP concentrations and source water as characterized by DOC, 8 samples collected over 16 weeks for Lake Ontario and Grand River (upstream and downstream), and 6 samples collected over 21 weeks for Otonabee River. Vertical bars represent ± one standard deviation (*n* = 2).

Variations in average ATP levels were also observed among virgin MPs conditioned in various water matrices with an approximate trend of Grand River > Otonabee River > Lake Ontario; however, only the comparison between the Grand River and Otonabee River was statistically significant (*p* < 0.05). This is further illustrated in Figure 2, which shows ATP associated with virgin PVC for each of the water matrices with respect to DOC. Other polymers displayed similar patterns (Figures S8–S11). Data in general cluster according to the water matrix. Overall, a positive trend between ATP and DOC was observed for all polymer types (Pearson correlation coefficient r = 0.51–0.69, *p* < 0.05). This was anticipated, as higher levels of DOC are an indication of more organic material available for biofilm growth. Similar trends were also observed when comparing ATP and UV254 (r = 0.53–0.68, *p* < 0.05), as well as turbidity (r = 0.62–0.71, *p* < 0.05) (Figures S12–S21). For reference, the historical nutrient data demonstrated a similar pattern with relatively higher total N and total *p* levels observed in the Grand River when compared to others (Table S1).

Additional parameters that may be useful when characterizing biofilm formation on polymers and other substrates include nutrient concentration, salinity, oxygen content, temperature, pH, light, and hydrodynamic conditions [17,53,57,58]. The current study did not observe any significant impact of temperature or pH on biofilm abundance (α = 0.05).

### 3.4. Impact of Weathering on Biofilm Growth

**Figure 3:**
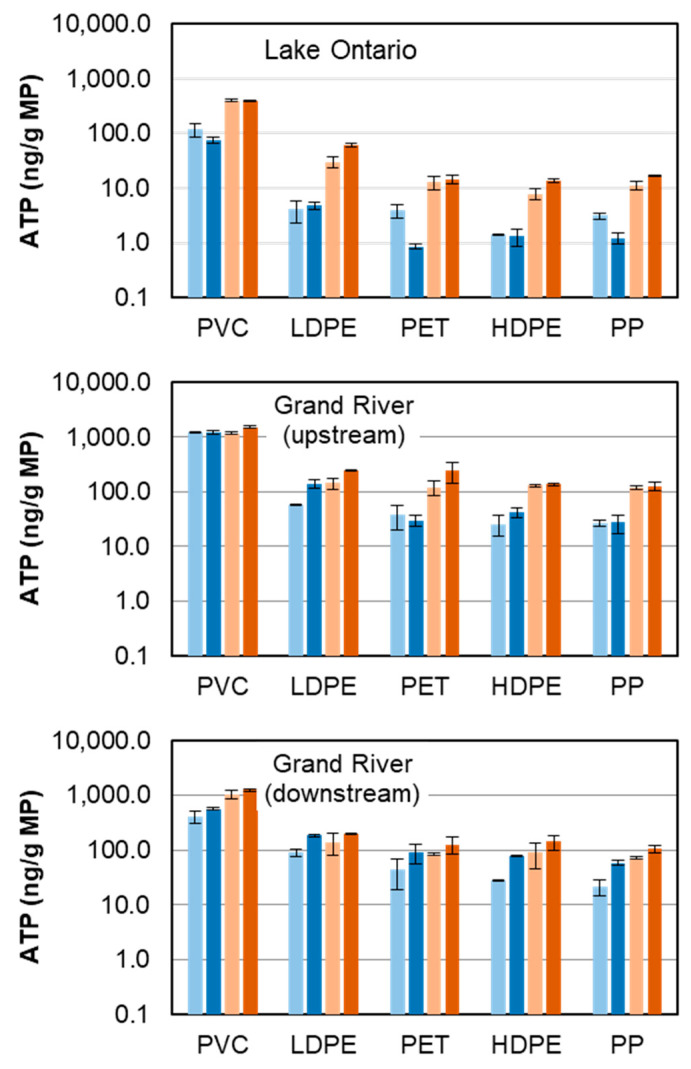
ATP concentrations associated with virgin and weathered polymers in three water matrices; “8 wk” or “16 wk” indicate exposure time in weeks, “V” = virgin, “W” = weathered; vertical bars indicate ± 1 standard deviation (Lake Ontario: DOC = 1.72–4.25 mg/L, temperature = 9.1–20.6 °C; Grand River (upstream): DOC = 5.31–13.15 mg/L, temperature = 5.9–28.1 °C; Grand River (downstream): DOC = 3.92–6.55 mg/L, temperature = 9.9–24.3 °C).

Virgin and weathered MPs were compared in terms of ATP concentrations following both 8 and 16 weeks of exposure (Figure 3). Most source water—exposure time combinations (Lake Ontario—8 and 16 weeks; Grand River (upstream)—8 and 16 weeks; Grand River (downstream)—8 weeks, and Grand River (downstream)—16 weeks) did not exhibit statistically significant differences (*p* < 0.05, two-tailed paired *t*-tests). Only differences observed for the Grand River (downstream) (both 8 weeks and 16 weeks) were statistically significant (*p* < 0.05); these were associated with the highest DOC concentration.

The most plausible explanation for differences in biofilm abundance when comparing weathered and virgin MPs was that the weathering process simply increased the available surface area for biofilm attachment [29,50,59]. This hypothesis was supported by elevated ATP levels observed following extended exposure times and/or in relatively high-DOC water only (Lake Ontario—16 weeks, Grand River (upstream)—16 weeks, Grand River (downstream)—8 and 16 weeks), suggesting that the limiting factor for biofilm growth was likely surface area rather than available microorganisms, nutrients, or oxygen in the source water [51].

Other factors not assessed in the current study may also contribute to differences in biofilms associated with weathered and virgin MPs. These could include the ability of weathered MPs to adsorb organic matter from the surrounding environment and create conditions that promote biofilm colonization [60]. As well, weathering may alter the polymer surface charge and subsequently enhance or impede biofilm attachment [28,61,62].

Another hypothesis is associated with decreased hydrophobicity on polymer surfaces, as weathering creates oxygen-containing polar functional groups, including carbonyl and carboxyl groups [34,45,63]. However, a definitive relation between hydrophobicity and biofilm growth has not been established. In theory, substrates with decreased hydrophobicity are less attractive to hydrophobic microorganisms in water [52,53]; conversely, elevated ATP levels were observed on weathered (less hydrophobic) MPs in the current study. Similarly, a study by others involving inoculated trials reported elevated bacterial abundance on eroded LDPE, HDPE, and polystyrene microplastics with lower hydrophobicity when compared to virgin samples as confirmed by contact angle measurements [28].

### 3.5. Microbial Community Composition on Virgin and Weathered Polymers

**Figure 4:**
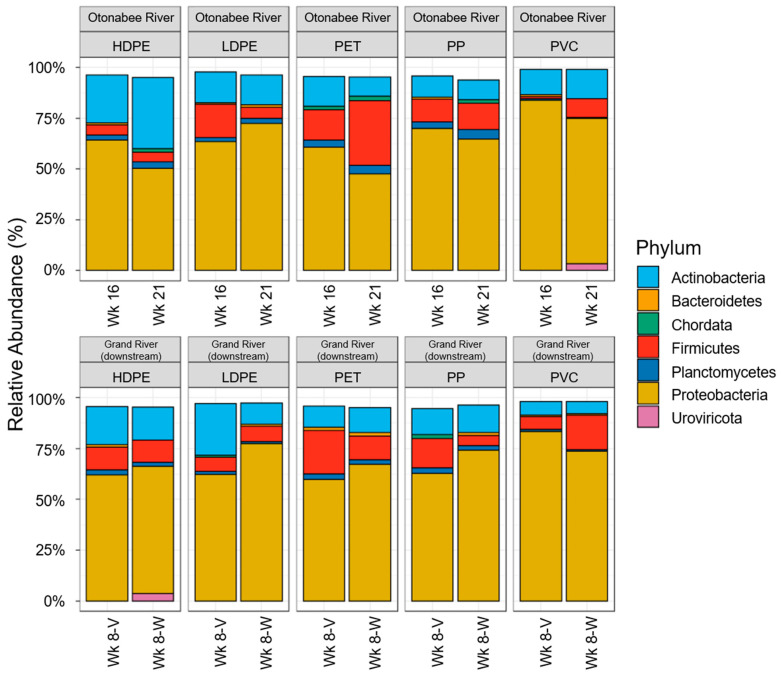
Phylum-level relative abundance within MP-associated biofilms. Selected polymer–source water combinations include 16- and 21-week virgin polymers and Otonabee River, and 8-week virgin and weathered polymers and Grand River (downstream).

Metagenomic analyses were conducted on 16- and 21-week virgin samples for the Otonabee River as well as 8-week virgin and weathered polymers for the Grand River (downstream) (Figure 4). Proteobacteria were the most abundant (48–84%), which were followed by Actinobacteria (6–35%) and Firmicutes (1–32%). The presence of bacteria in these phyla is not surprising, as they are commonly observed in freshwaters [64]. Similar results have been reported by others. Wu et al. reported 60–77% Proteobacteria and 6–14% Firmicutes within microbial communities on PVC incubated in river water [15], Miao et al. reported 40–73% Proteobacteria in biofilm attached to PE and PP incubated in lake water [16], and González-Pleiter et al. observed 76.1% Proteobacteria and 3.1% Actinobacteria with PE incubated in lake water [49]. Song et al. also reported a high abundance of Proteobacteria (26–46%) on PE and PET pellets incubated in tap water or lake water as well as 26–39% Planctomycetes in contrast to the <10% abundance observed in this study, which may be attributed to variations in the source waters employed [14].

Although exposure time did not exhibit a discernable impact on microbial community structure, a trend was observed when comparing virgin and weathered MPs. With the exception of PVC, all polymers showed increases in relative abundance of Proteobacteria on weathered polymers, ranging from 1% (HDPE) to 15% (LDPE). Considering the observed dominance of Proteobacteria, these increases in part explained the elevated ATP levels observed for weathered Grand River (downstream) polymers.

**Figure 5:**
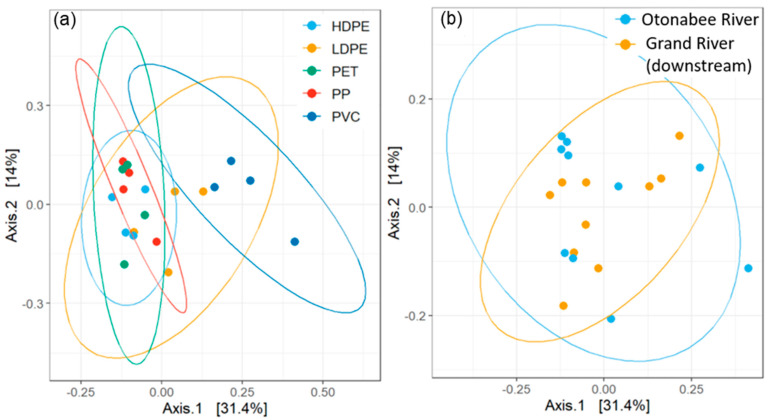
PCoA plots of biofilm communities based on Bray–Curtis dissimilarity, ellipses created at 95% confidence based on (**a**) polymer type and (**b**) source water.

To further examine the phylogenetic diversity of biofilm samples, principal coordinate analysis (PCoA) plots were generated on the basis of measured Bray–Curtis dissimilarities. Ellipses were plotted corresponding to 95% confidence levels based on different group characteristics, including polymer type (Figure 5a) and source water (Figure 5b), as well as weathering condition and exposure time (Figure S22). Qualitatively, some visible differentiation was observed among polymer types: principal component 1 (Axis. 1) explained 31.4% of the divergence in microbial community diversity along which the PVC-associated cluster was clearly separated from others. In addition, along the same axis, clusters of HDPE, PET, and PP largely overlap, with the LDPE cluster primarily spread between two groups. This was consistent with the ATP level trend observed among polymer types and indicated that observed differences in biofilm abundance could at least be partially attributed to the enrichment of various microorganisms with respect to different polymers. In contrast, Song et al. assessed biofilm growth on microplastics submerged in lake and tap water over 30 d and reported that the original bacterial communities in each matrix were the primary determinants of microplastic-associated biofilm composition [14].

Permutational multivariate analysis of variance (PERMANOVA) based on Bray–Curtis dissimilarities was conducted to quantify the variation in microbial community structures among different groups. Significant diversity was observed when comparing samples of different polymer types (*p* < 0.05) as well as a lack thereof associated with weathering or exposure time (*α* = 0.05). Differentiation due to water matrix (Otonabee River vs. Grand River), although not observed in PCoA, was also statistically significant (*p* < 0.05). This discrepancy indicated that potentially, the result could be statistically significant but not biologically meaningful. Overall, the impact of these parameters on biofilm composition largely remained inconclusive, which was likely due to the complexity of the biofilm formation process. A previous study observed significant phylogenetic differences when comparing PE and PP to natural materials (cobblestone and wood) but not between polymers [16]. The impact of environmental influences on biofilm was anticipated, and it has been reported as the primary factor in biofilm formation on polymers [17]. It should also be noted that different source waters typically contain distinct microbial communities. Weathering increased biofilm abundance for the site with the highest DOC (indicated by ATP) but did not significantly impact microbial community structure, which further supports the hypothesis that elevated biofilm abundance on weathered polymers is due to increases in surface area.

### 3.6. Pathogens Present in Microplastic-Associated Biofilms

Potential pathogens were identified at the species level. *Salmonella enterica* and *Escherichia coli*, both pathogen-containing species known to cause illness including typhoid and diarrhea [65,66], were observed with >0.1% relative abundance in biofilms on all analyzed polymers (Figure S23). Relative abundances of *S. enterica* ranged from 0.4% to 13.7%, with the highest being associated with PP. The relative abundance of *E. coli* was typically lower when compared to *S. enterica*, contributing only up to 1.2% in all samples analyzed except for Grand River (downstream) virgin LDPE, in which a relative abundance of 12.4% was observed.

A trend between relative abundances and polymer types, regardless of species, was observed for Otonabee River water: PP ≈ HDPE ≈ PET > LDPE > PVC, which represented the reverse of the ATP trend. This indicated that the high ATP levels associated with PVC and LDPE were not proportionally contributed by these potential pathogens but rather by other species. However, following normalization to ATP levels observed on the same polymers, the estimated amounts of *S. enterica* and *E. coli* associated with PVC remained up to 17 times higher when compared to other polymer types. No discernible pattern was observed regarding polymer types when examining Grand River (downstream) water; however, weathered polymers consistently showed an elevated relative abundance of *S. enterica* (up to 7.6% higher than virgin counterparts). This trend was in agreement with the elevated relative abundance of Proteobacteria, as *S. enterica* is a species within this phylum. This observation is of particular concern, as it potentially indicates the selective enrichment of *S. enterica* in biofilm associated with weathered polymers.

It should be noted that the interpretation of *S. enterica* and *E. coli* presence within biofilm should be considered with caution, since only specific subspecies of *S. enterica* and strains of *E. coli* pose health risks [65]. Other studies have reported potential human pathogens in MP-associated biofilms in freshwaters, including species of *Pseudomonas* [15,26], *Arcobacter* [23], and *Vibrio* [17], some of which were also observed in the current study albeit at extremely low relative abundances (<0.03%).

## 4. Conclusions

The growth of biofilm on both virgin and weathered MPs was evident regardless of water matrix or polymer type. Among the polymers examined, PVC was of particular interest, as it was associated with ATP levels that were 6 to 12 times higher when compared to other polymer types. While weathered polymers may better represent those found in the environment, statistically significant differences in ATP concentration and biofilm composition were not observed when compared to virgin polymers. Polymer type was the primary determinant of biofilm growth, while weathering resulted in increased ATP for a source water with high DOC (>4 mg/L). Pathogen-containing species including Salmonella enterica and Escherichia coli were observed with 0.1–13.7% relative abundance on all polymers, with *S. enterica* appearing to be selectively enriched on weathered MPs when considering specific freshwater matrices. The current study focused on MP-associated biofilm growth in WTP source waters only; the impact of downstream drinking water treatment processes was not considered but should remain an important topic for future research.

## Data Availability

Data are available upon request.

## Supplementary Materials

The following supporting information can be downloaded at: https://www.mdpi.com/article/10.3390/toxics11120987/s1. Detailed description of the passive flow-through system, weathering apparatus, and bond index calculation; discussion regarding the cyclic pattern of MP weathering; water quality parameters during in situ trials; description of polymer pellets; surface roughness of virgin and weathered MPs; microscope images of weathered MPs; FTIR and O/C ratio results; trends between ATP and water quality parameters; additional PCoA plots; and relative abundance of *Salmonella enterica* and *Escherichia coli* in biofilm. Sequence data generated in this study are publicly available under NCBI BioProject number PRJNA849345 [34,36,46,67,68,69,70,71,72,73,74,75,76,77,78].
