# Supplementary material for: Assessment of Biofilm Growth on Microplastics in Freshwaters Using a Passive Flow-Through System
Source: Toxics. 2023 Dec 5;11(12):987. doi: 10.3390/toxics11120987 (PMC10748376; doi:10.3390/toxics11120987)
Supplement: Supplementary file 1 [file toxics-11-00987-s001.zip › toxics-2721596-supplementary.pdf]

toxics

Supplementary Materials: Assessment of Biofilm Growth on Microplastics in Freshwaters using a Passive Flow-Through System

Chengyang Jiang, Husein Almuhtaram, Michael J. McKie and Robert C. Andrews

1. Detailed Description of Passive Flow-Through System

The "passive flow-through system" used in all trials to expose different polymers to source waters was designed and custom built in-house (Figure S1). It consisted of a stainless steel cylindrical vessel, 50 cm in inner diameter and 50 cm in height, with inlet and outlet ports at the bottom and top, respectively, as well as at opposite sides to minimize short-circuiting. Three perforated stainless steel plates (0.25" diameter, 58% open, approximately 6 cm between plates) were included where a bottom plate served as a baffle to assist in providing laminar flow; two upper plates were used to support polymers (contained in stainless steel mesh bags). A motor-powered (< 10 RPM) propeller-shaped blade provided mixing in both tangential (horizontal) and axial (vertical) directions. Polymer pellets (3–5 g, both virgin and weathered) were held in 7 cm × 7 cm bags, prepared from 100 μm stainless-steel mesh (McMaster-Carr, Robbinsville, NJ) to allow ambient water to pass through while retaining the particles.

2. Detailed Description of Weathering Apparatus

The weathering apparatus (Figure S2) was adapted from an existing study, which considers both hydrolytic and photooxidative weathering of MPs and avoids the use of unrealistic exposure conditions [36]. The system was subsequently modified to mimic weathering conditions in North American freshwaters.

In brief, 1 L borosilicate glass cylinders contained approximately 20 g of a specific polymer type (HDPE, LDPE, PP, PET, or PVC), 750 mL of Elix® water, and 100 mL of silica sand. Constant agitation and aeration were achieved with aeration. A metal halide lamp (Daylight Blue 600W MH, Hortilux, Mentor, OH) was used to simulate sunlight, the output of which was adjusted using a dimmable ballast such that the water received radiation exposure representative of in situ conditions in North America. Individual components are described in the following.

2.1. System Components

1L borosilicate graduated cylinders were used, as previous studies have shown that borosilicate glass does not obstruct UV radiation [34,67]. Each 1L cylinder contained 750 mL of Elix® water which was refilled every other day to compensate for evaporation losses. All weathering was conducted at room temperature (22–25 °C). Similar water temperatures have been observed in the Great Lakes [68], although mainly during the summer season. The conditions employed is considered realistic, especially when compared to the elevated temperatures reported in previous weathering studies which reached 60 °C [69,70].

Approximately 100 mL of sand was added to each 1L glass cylinder to aid in mechanical abrasion. Standard Filter Sand (Anthrafilter Media & Coal Ltd., Brantford, ON) was used with an effective size of 0.46 mm (range 0.15–0.85 mm). To avoid any initial biological contamination, the sand was initially heated at 400 °C for at least 12 h prior to use. In total, 8 weathering cylinders were operated simultaneously under the same conditions, such that an appropriate mass of polymers could be produced for use in all trials.

2.2. Agitation and aeration

Diffused air in combination with sand was used to create mechanical abrasion (by the movement of both water and suspended particles) of polymer pellets. The diffused air system consisted of an 8-outlet 60W 70 L/min air pump (Hydrofarm, Petaluma, CA) connected to stone aerators placed at the bottom of each cylinder. Air flow input to each diffusor was adjusted using a valve such that the contents of each cylinder were reasonably disturbed without causing water to overflow.

In addition to providing mechanical abrasion, aeration allowed simulation of upper-level oxygen-rich waters where most floating polymers are reported [67,71].

2.3. Radiation

With respect to the radiation source used for photooxidative weathering of plastics, Andrade et al. compared a variety of lighting equipment and recommended the use of metal halide lamps that "offered spectra which highly resemble the solar spectrum (at ground level)" [36].

In our system a Daylight Blue 600W MH lamp ("Daylight Blue") was selected. Figure S3 demonstrates the comparison between the emission profile (relative energy distribution through the wavelengths) of the Daylight Blue and the Powerstar used by Andrade et al., with the solar radiation baseline as the background. Since absolute intensity may vary depending on the power output of the lamp, the shape of the spectra indicates the relative energy distribution which should be the focus when interpreting. The Daylight Blue lamp (Figure S3) offers a spectrum which is similar to the Powerstar and solar radiation, with the exception of a peak at approximately 540 nm. This peak is likely due to metal components used in metal halide lamps, in this case, thallium which emits at 535 nm [72]. Similar peaks were observed from the Powerstar spectra.

During weathering trials, the UV lamp was placed approximately 50 cm from the top of the weathering cylinders to ensure even radiation. Within each system, the positions of weathering cylinders were swapped frequently to ensure uniform exposure.

As recommended by Andrade et al. [36], both overall illuminance and UV irradiance were validated to ensure that the system reasonably mimicked real-life conditions. Illuminance was measured using a Dual-Range light meter (Traceable, Webster, TX), and converted to irradiance using a simplified conversion factor for sunlight [73]. UV irradiance was measured using a UV513AB UVA/B light meter (General Tools & Instruments, New York, NY). Since this instrument measures combined irradiance associated with UV-A and UV-B (280–400 nm), an approximate UV-B (315 nm) value was extrapolated according to the lamp spectra and instrument calibration curve. In addition, UV irradiance was measured both in front of and behind individual weathering cylinders, with respect to the lamp, such that an approximate UV penetration rate in water could be calculated. Measurements were taken regularly throughout the weathering trials to account for any degradation of the UV lamps following extended periods of usage. Results were compared to actual conditions in Ontario, Canada as well as the values reported by Andrade et al. (Table S4) [36]. The radiation conditions reasonably simulated those in North America, in contrast to the elevated intensities reported in some previous weathering studies which could reach over 700 $W \cdot m^{-2}$ in irradiance [74].

3. Detailed Description of Bond Index Calculation

The formula used to calculate the carbonyl index is presented below to serve as an example:

$$\text{Carbonyl Index} = A_{1750\text{-}1690} - A^{0}_{1750\text{-}1690} \quad \text{(S1)}$$

where $A_{1750\text{-}1690}$ is the normalized peak area of the carbonyl groups of weathered polymers and $A^{0}_{1750\text{-}1690}$ is the corresponding peak area of virgin polymers. The calculation of vinyl, hydroxyl, and carbon-oxygen indices shares the same form, with only different wave numbers being assigned to specific functional groups, i.e., 1660–1616 cm^{-1} for

vinyl groups, 3650–3150 cm^{-1} for hydroxyl groups, and 1200–1000 cm^{-1} for carbon-oxygen groups. The combined C=X index was calculated within the range of 1810–1550 cm^{-1}.

4. Discussion Regarding the Cyclic Pattern of MP Weathering

Despite the varying responses of polymers to weathering, as characterized by FTIR (Figure S5), a fluctuating trend was observed for many of the indices across all polymers. Similar non-monotonous trends have been reported by others for PE [34,46], PP [36,46,75], and PVC [76]. These fluctuations may potentially be attributed to less weathered interior polymer being exposed as deterioration takes place at the surface, which in itself could be a result of polymer chain scission and dissolution into water [36], an increase of material brittleness causing fractures and holes [34], formation of volatile compounds on the polymer surfaces [46], or a combination of the above. As the newly exposed surface continued to deteriorate under weathering, a cyclic process was observed (Figure S7).

Table S1. Historical data of total N and P in source waters.

	Average total N (mg/L)	Total N range (mg/L)	Average total P (mg/L)	Total P range (mg/L)
Lake Ontario	0.4 ± 0.1	0.3 – 0.5	0.005 ± 0.005	0.005 – 0.01
Otonabee River	0.3 ± 0.1	0.1 – 0.5	0.01 ± 0.008	0.01 – 0.04
Grand River (upstream)	N/A	N/A	N/A	N/A
Grand River (downstream)	4.6 ± 1.9	0.2 – 7.8	0.014 ± 0.019	0.02 – 0.05

Results were obtained from the Drinking Water Surveillance Program (DWSP) 2018-2020 [77]. Data was not available for Grand River (upstream), but can be assumed similar to that of Grand River (downstream).

Table S2. Temperature, pH, turbidity, UV254, and DOC of source waters during in situ trials.

	Temperature (°C)	pH	Turbidity (NTU)	UV254	DOC (ppm)
Lake Ontario	9.1 – 20.6	7.4 – 8.5	0.26 – 0.66	0.013 – 0.092	1.72 – 4.25
Otonabee River	22.1 – 27.3	7.3 – 7.9	0.52 – 1.20	0.124 – 0.185	5.52 – 5.68
Grand River (upstream)	6.5 – 28.1	7.8 – 8.2	0.77 – 13.80	0.197 – 0.555	5.31 – 13.15
Grand River (downstream)	9.9 – 24.3	7.4 – 8.4	1.97 – 5.40	0.146 – 0.310	3.92 – 6.55

Table S3. Description of polymers.

	Colour	Shape	Average Diameter (mm)
HDPE	Clear, translucent	Sphere	3.5
LDPE	Clear, translucent	Sphere	3.5
PP	Grey	Sphere	3.5
PET	White	Sphere-like cylinder	3.0
PVC	Clear, translucent	Sphere	3.5

Table S4. Comparison of radiation intensities.

	Current System	Ontario, Canada	Andrade et al. (2019)
Overall irradiance ($W{\cdot}m^{-2}$)	98.8 – 135.1	100 – 240	96.4
UVA/B ($W{\cdot}m^{-2}$)	10.60 – 14.23	10.12 – 15.25	
UVB ($W{\cdot}m^{-2}$)	0.76 – 1.02		0.60
UV penetration in water	47.9%		41.7%

Overall irradiance in Ontario was published by Natural Resources Canada [78]. The UVA/B for Ontario was measured in Toronto, ON, on a mostly sunny day in October.

Table S5. Mean surface roughness of virgin and weathered microplastics.

Polymer Type	Mean Roughness (µm)		p value
	Virgin	Weathered	
**HDPE**	1.15	1.29	0.57
LDPE	1.56	1.29	0.25
PET	4.05	3.83	0.83
PP	4.17	6.38	0.03 *
PVC	2.55	3.03	0.48

* $p < 0.05$, suggesting significant difference between surface roughness of virgin and weathered samples.

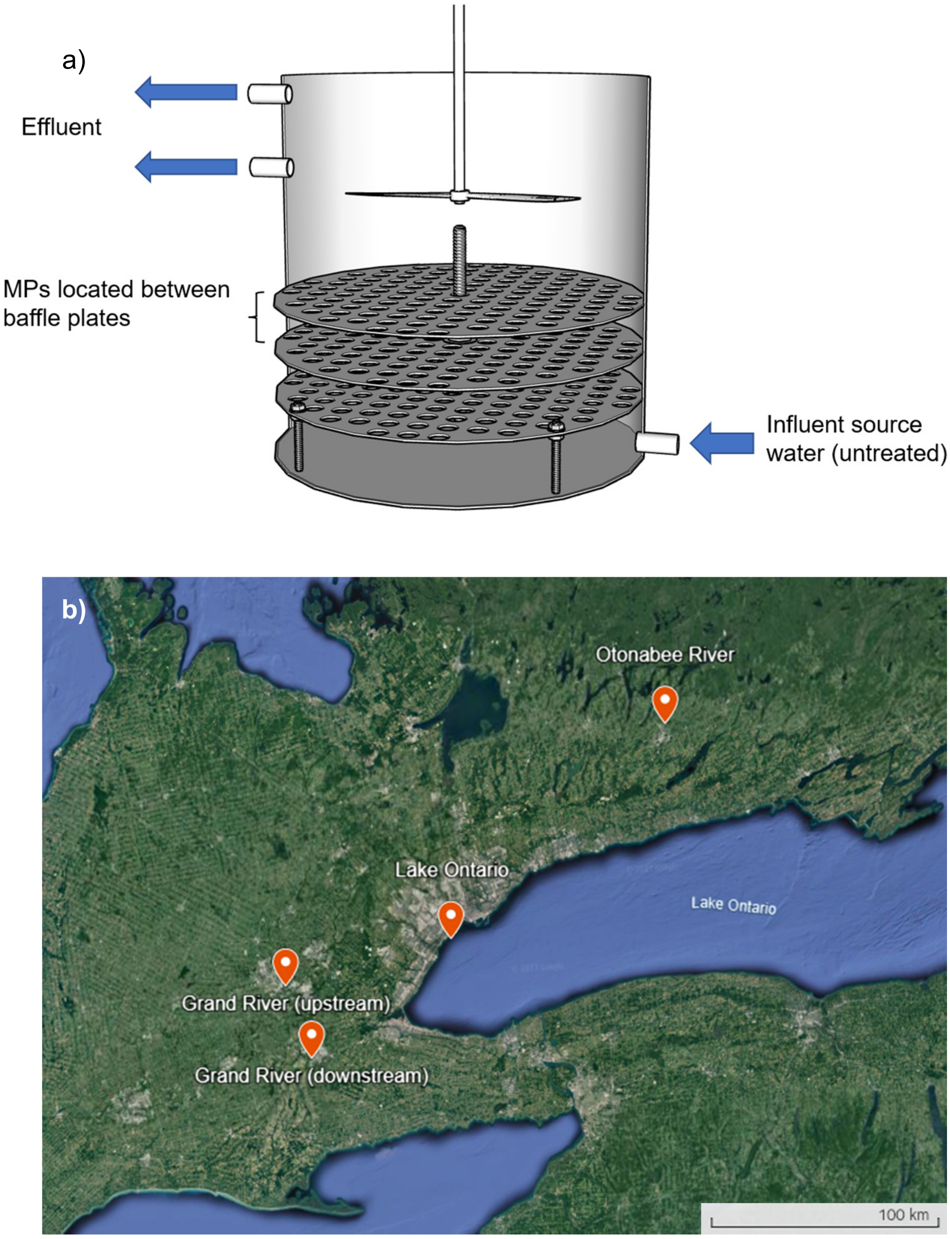

Figure S1. (a) Diagram of the passive flow-through system used for passive biofilm development and (b) locations where the system was deployed in the province of Ontario, Canada.

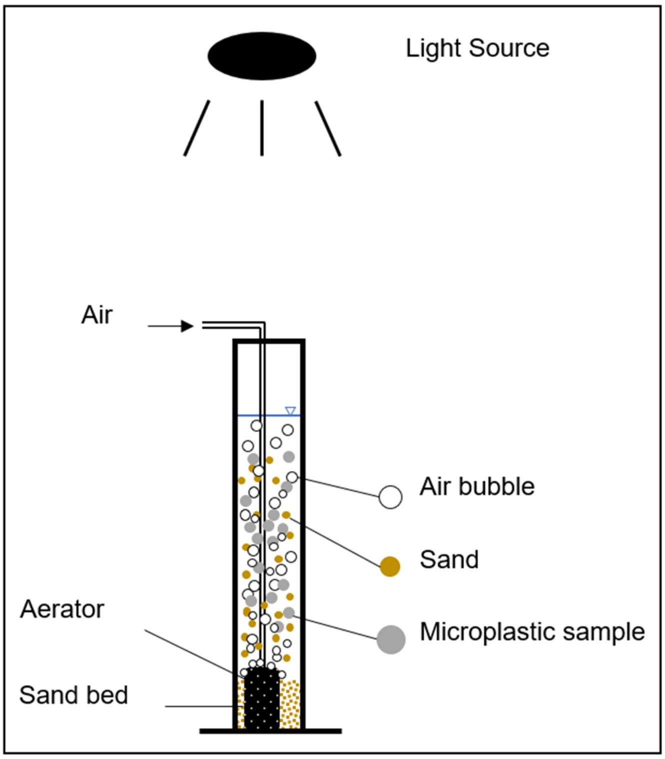

Figure S2. Diagram of apparatus used for MP weathering.

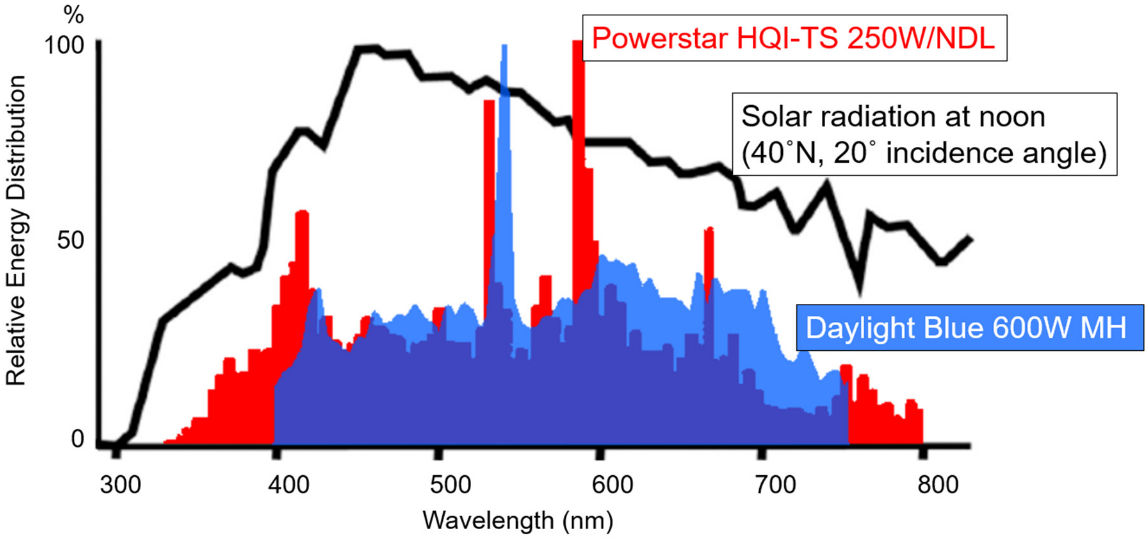

Figure S3. Comparison between lamp emission and solar radiation spectrum. The reference solar radiation and the Powerstar spectra are adopted from Andrade et al. [36]. Spectrum of Daylight Blue is available from 400 to 750 nm.

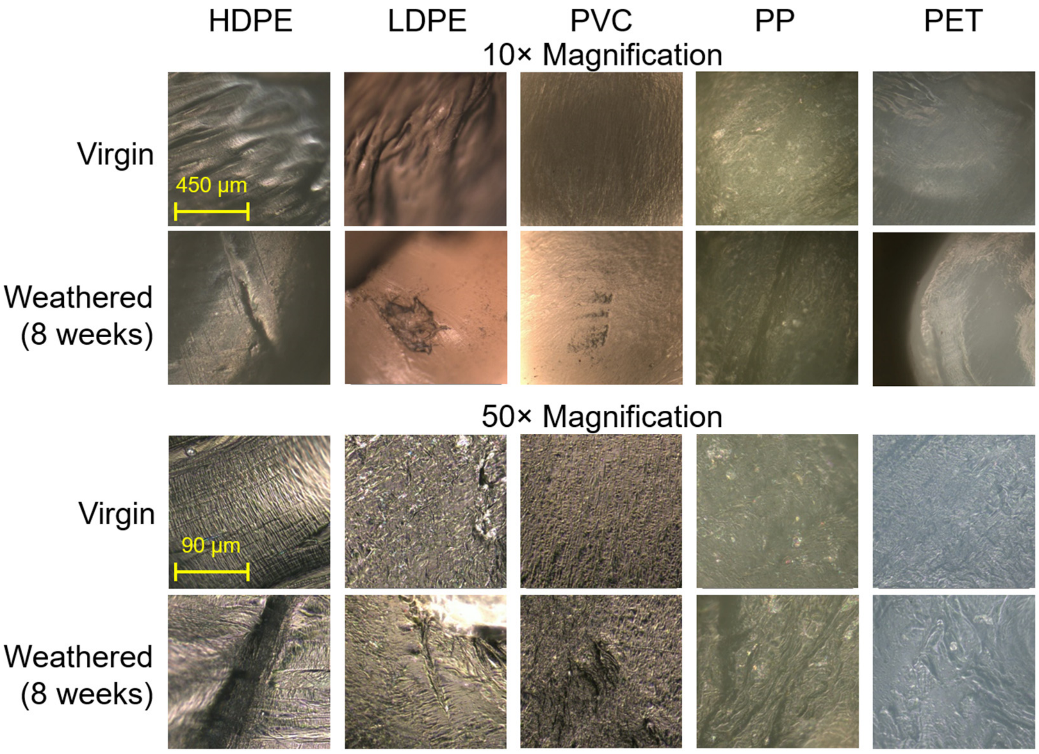

Figure S4. Microscope images obtained for virgin polymers and following 8 weeks of weathering.

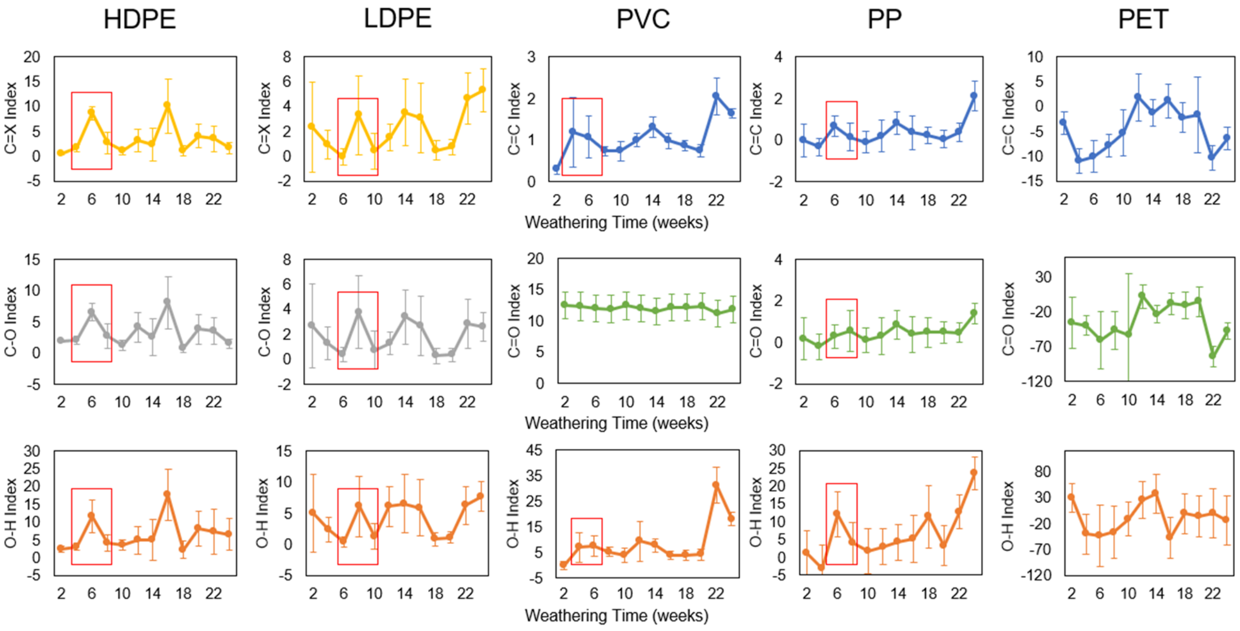

Figure S5. Bond indices for all polymer types following 24 weeks of weathering, vertical bars indicate ± 1 standard deviation, areas highlighted in red represent evidence of oxidation following 6–8 weeks of weathering.

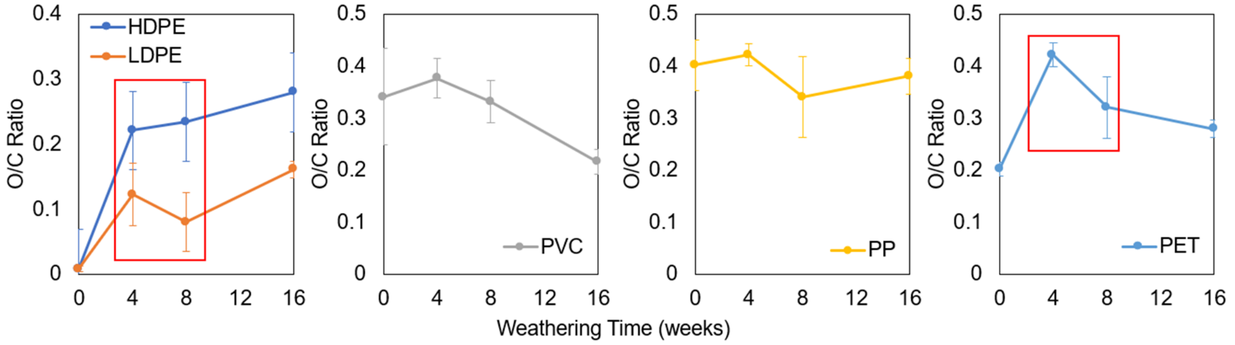

Figure S6. Oxygen/carbon (O/C) ratios for all polymer types following 16 weeks of weathering, vertical bars indicate ± 1 standard deviation, areas highlighted in red boxes represent evidence of oxidation following 4–8 weeks of weathering.

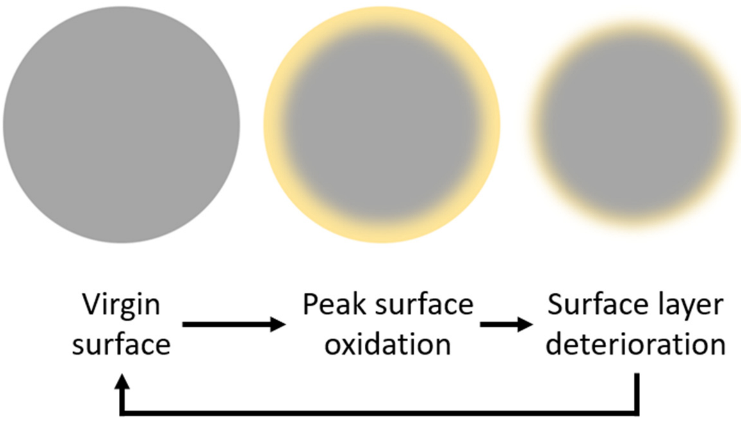

Figure S7. Hypothetic model of the cyclic weathering process of polymers (not to scale).

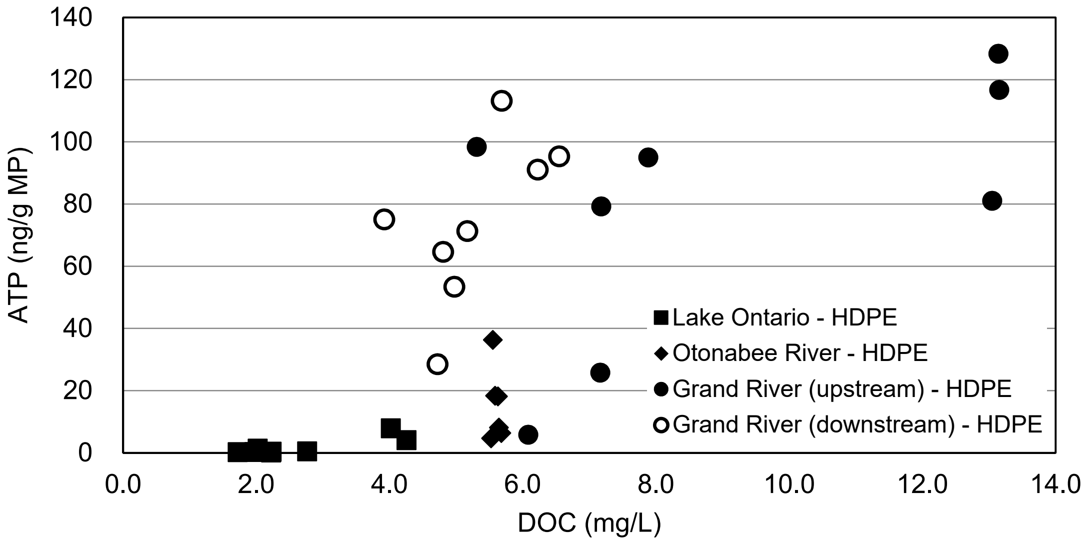

Figure S8. Correlation between HDPE-associated ATP and source water as characterized by DOC. 8 samples collected over 16 weeks for Lake Ontario, Grand River (upstream), and Grand River (downstream), 6 samples collected over 21 weeks for Otonabee River.

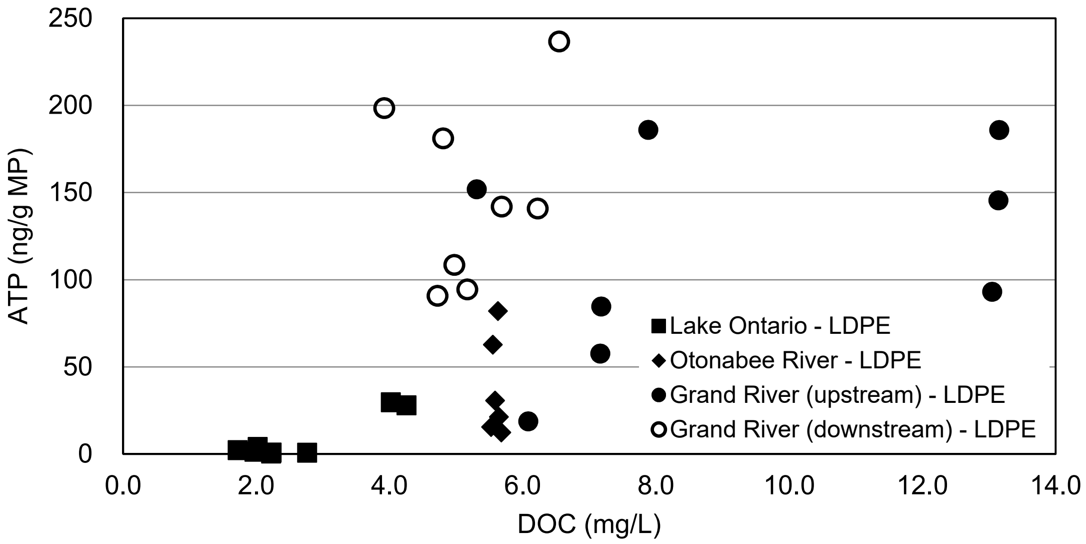

Figure S9. Correlation between LDPE-associated ATP and source water as characterized by DOC. 8 samples collected over 16 weeks for Lake Ontario, Grand River (upstream), and Grand River (downstream), 6 samples collected over 21 weeks for Otonabee River.

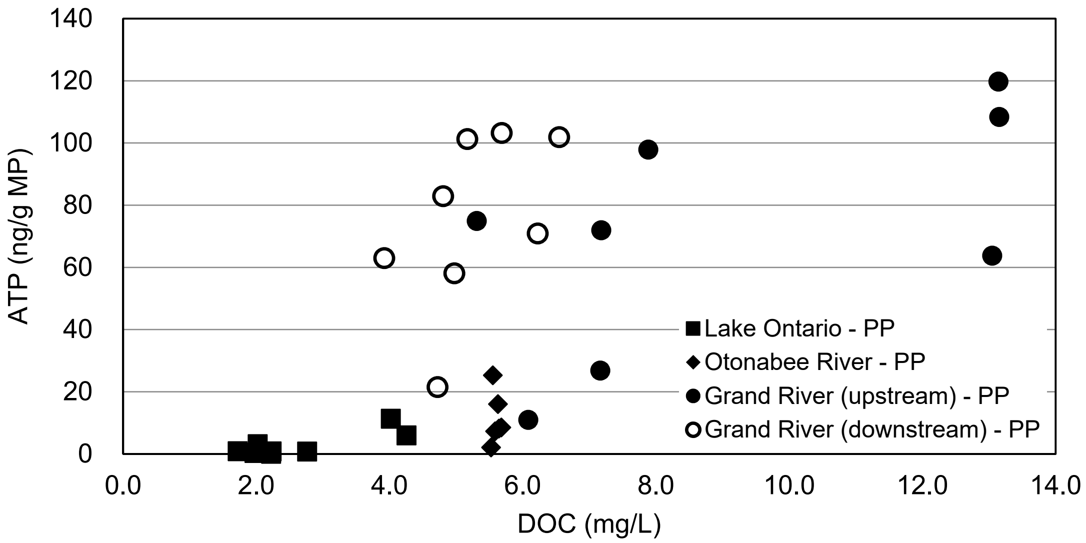

Figure S10. Correlation between PP-associated ATP and source water as characterized by DOC. 8 samples collected over 16 weeks for Lake Ontario, Grand River (upstream), and Grand River (downstream), 6 samples collected over 21 weeks for Otonabee River.

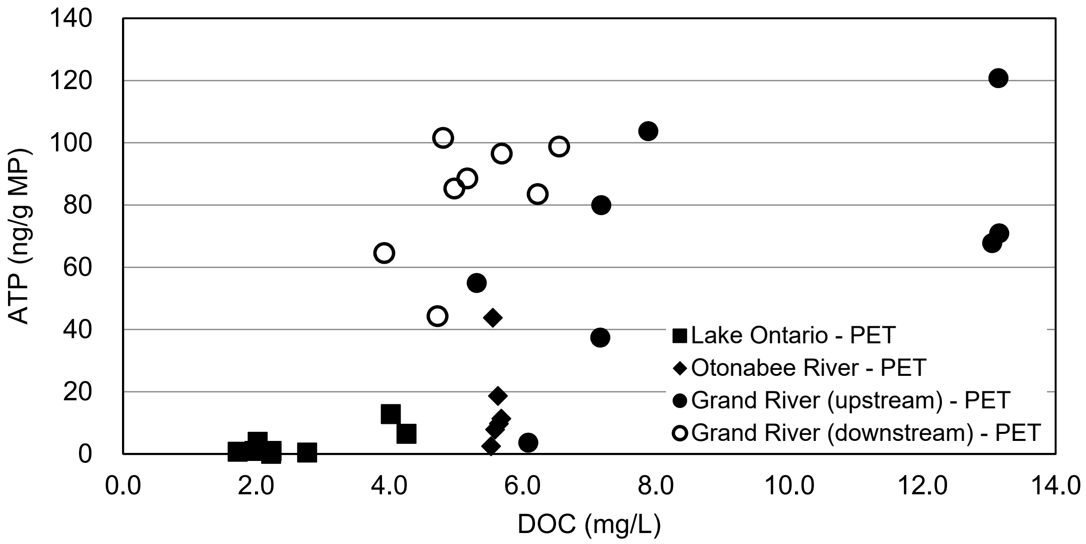

Figure S11. Correlation between PET-associated ATP and source water as characterized by DOC. 8 samples collected over 16 weeks for Lake Ontario, Grand River (upstream), and Grand River (downstream), 6 samples collected over 21 weeks for Otonabee River.

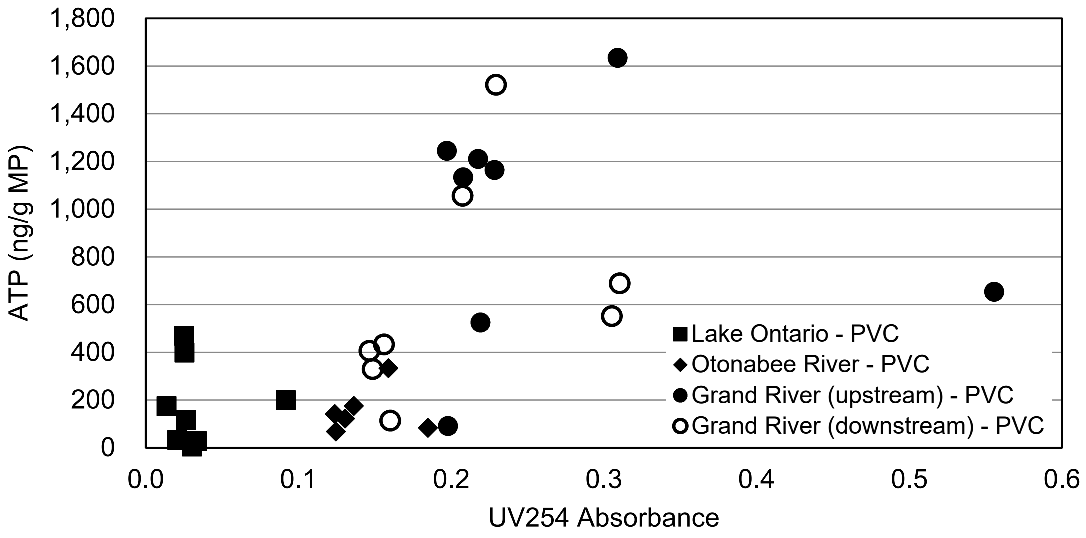

Figure S12. Correlation between PVC-associated ATP and source water as characterized by UV254. 8 samples collected over 16 weeks for Lake Ontario, Grand River (upstream), and Grand River (downstream), 6 samples collected over 21 weeks for Otonabee River.

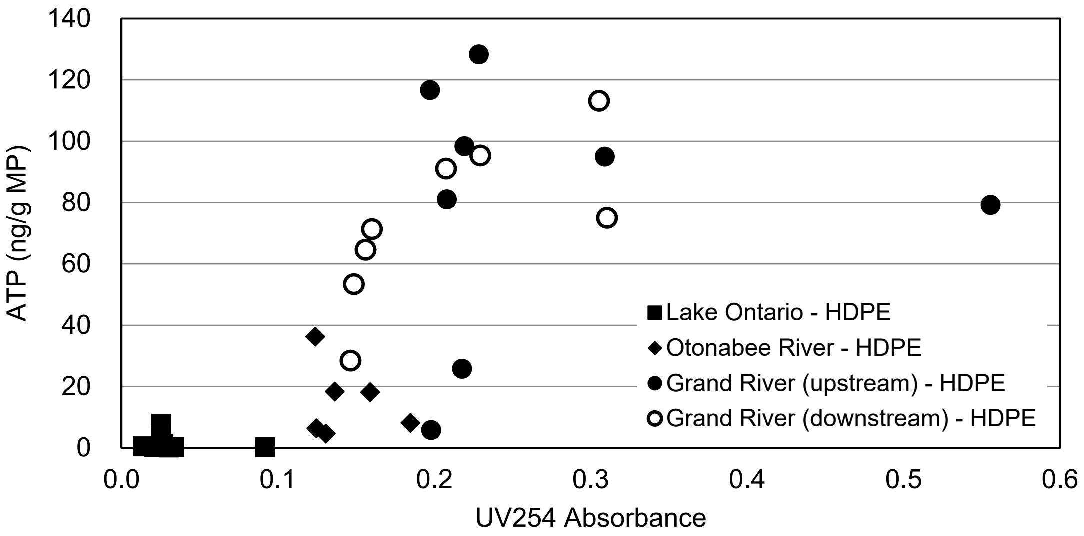

Figure S13. Correlation between HDPE-associated ATP and source water as characterized by UV254. 8 samples collected over 16 weeks for Lake Ontario, Grand River (upstream), and Grand River (downstream), 6 samples collected over 21 weeks for Otonabee River.

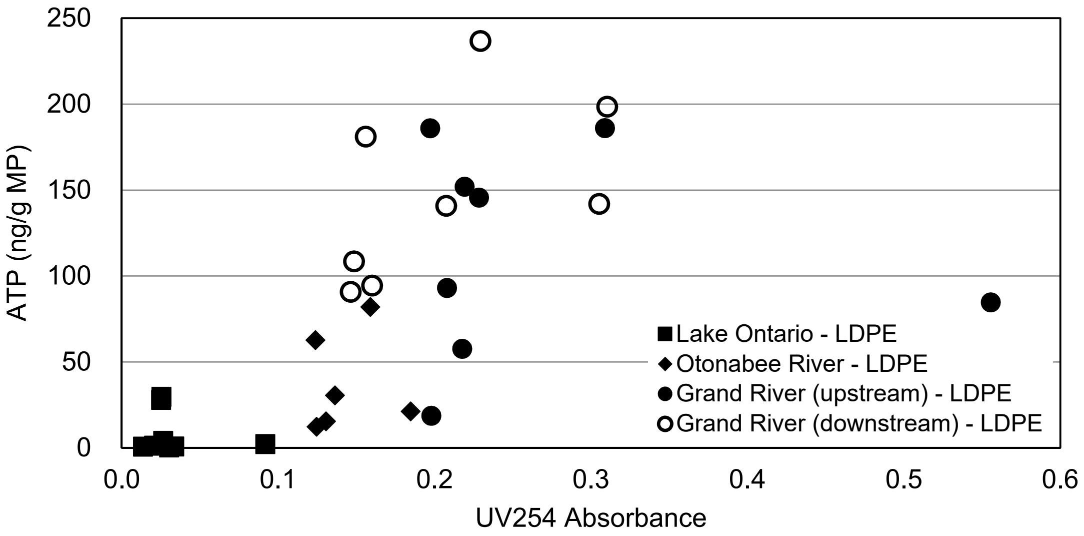

Figure S14. Correlation between LDPE-associated ATP and source water as characterized by UV254. 8 samples collected over 16 weeks for Lake Ontario, Grand River (upstream), and Grand River (downstream), 6 samples collected over 21 weeks for Otonabee River.

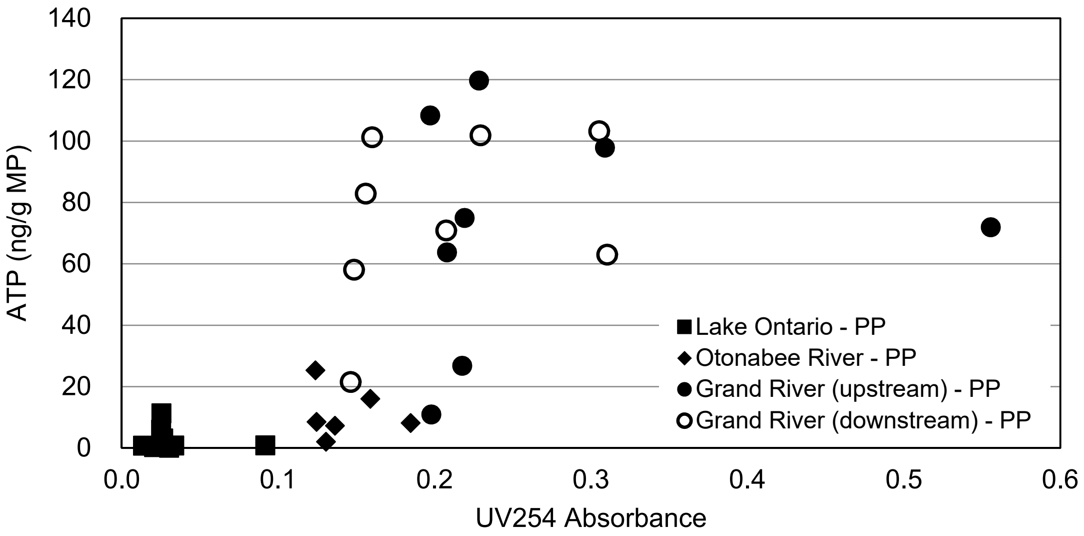

Figure S15. Correlation between PP-associated ATP and source water as characterized by UV254. 8 samples collected over 16 weeks for Lake Ontario, Grand River (upstream), and Grand River (downstream), 6 samples collected over 21 weeks for Otonabee River.

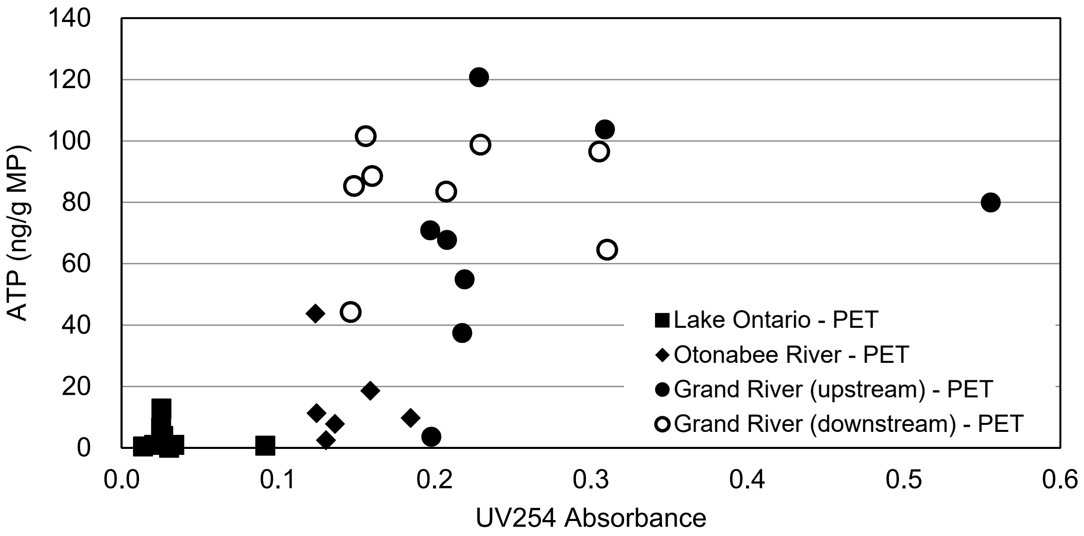

Figure S16. Correlation between PET-associated ATP and source water as characterized by UV254. 8 samples collected over 16 weeks for Lake Ontario, Grand River (upstream), and Grand River (downstream), 6 samples collected over 21 weeks for Otonabee River.

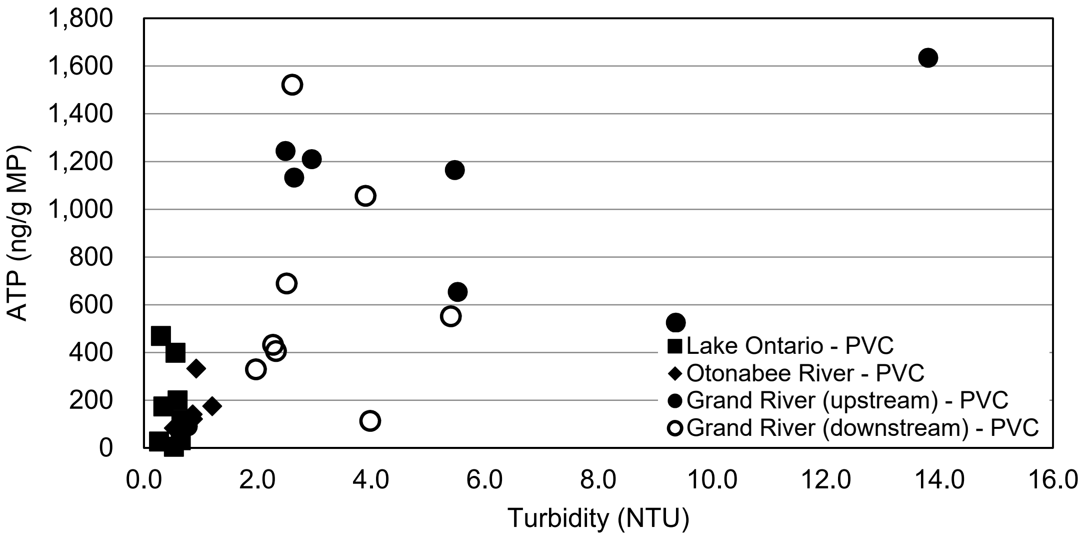

Figure S17. Correlation between PVC-associated ATP and source water as characterized by turbidity. 8 samples collected over 16 weeks for Lake Ontario, Grand River (upstream), and Grand River (downstream), 6 samples collected over 21 weeks for Otonabee River.

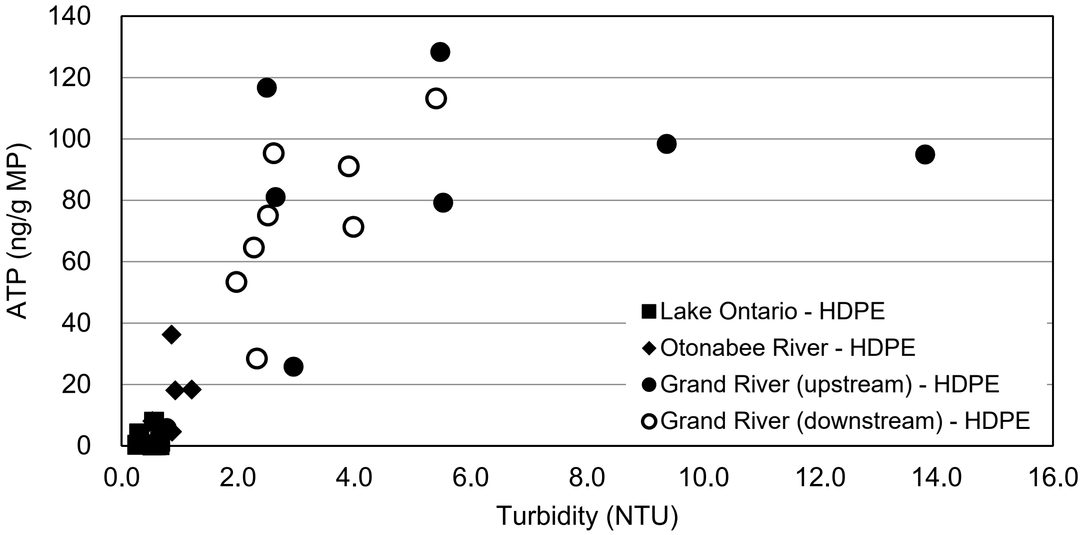

Figure S18. Correlation between HDPE-associated ATP and source water as characterized by turbidity. 8 samples collected over 16 weeks for Lake Ontario, Grand River (upstream), and Grand River (downstream), 6 samples collected over 21 weeks for Otonabee River.

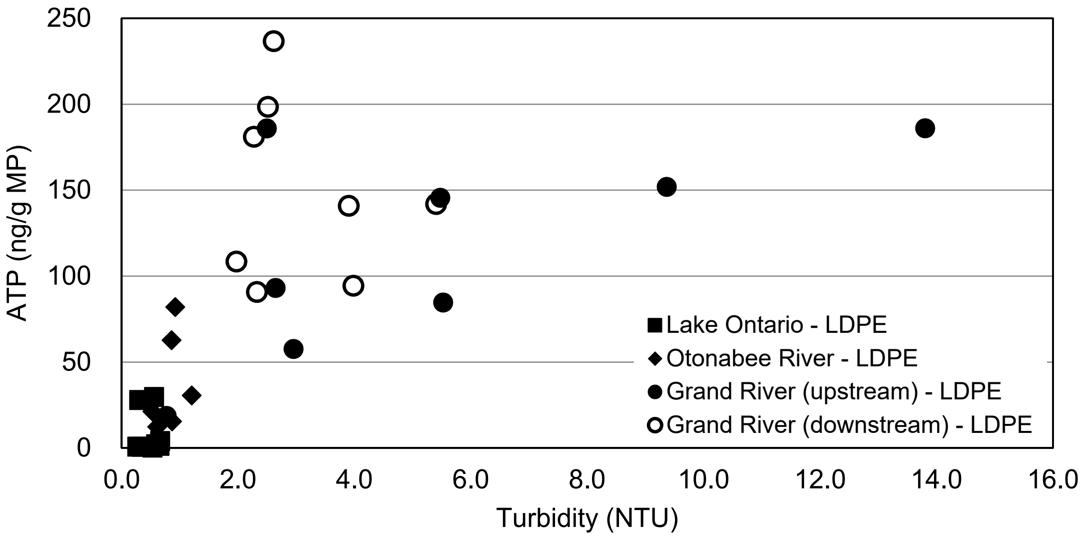

Figure S19. Correlation between LDPE-associated ATP and source water as characterized by turbidity. 8 samples collected over 16 weeks for Lake Ontario, Grand River (upstream), and Grand River (downstream), 6 samples collected over 21 weeks for Otonabee River.

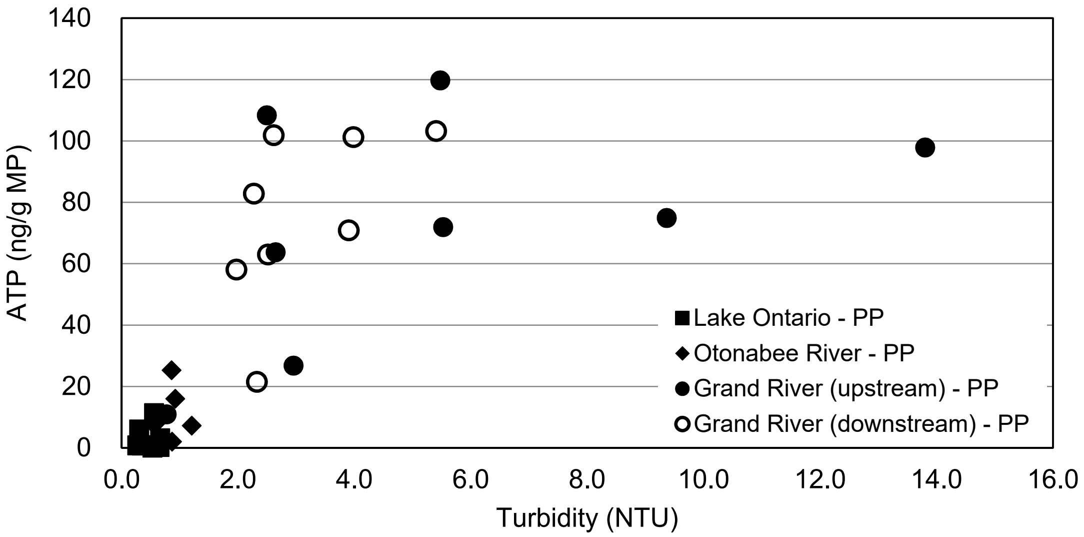

Figure S20. Correlation between PP-associated ATP and source water as characterized by turbidity. 8 samples collected over 16 weeks for Lake Ontario, Grand River (upstream), and Grand River (downstream), 6 samples collected over 21 weeks for Otonabee River.

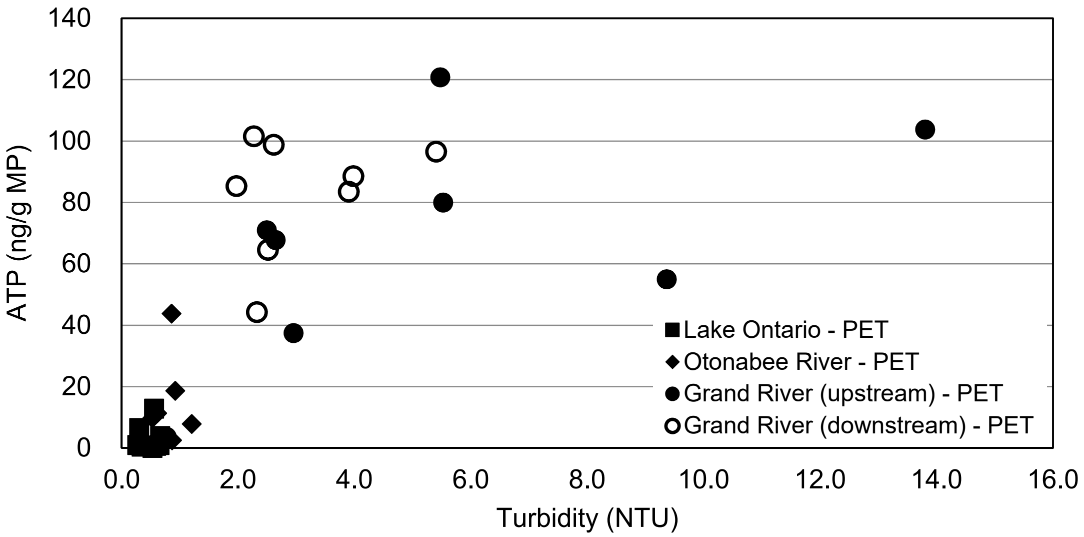

Figure S21. Correlation between PET-associated ATP and source water as characterized by turbidity. 8 samples collected over 16 weeks for Lake Ontario, Grand River (upstream), and Grand River (downstream), 6 samples collected over 21 weeks for Otonabee River.

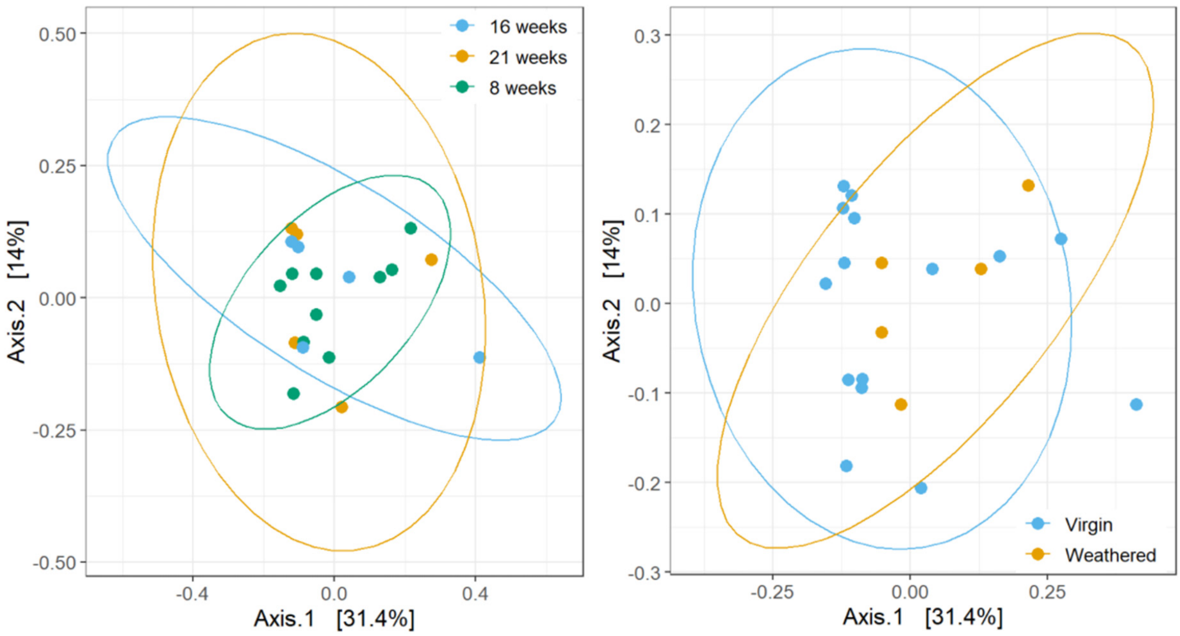

Figure S22. PCoA plots of biofilm communities based on Bray-Curtis dissimilarity. Ellipses created at 95% confidence based on exposure time (left) and weathering condition (right).

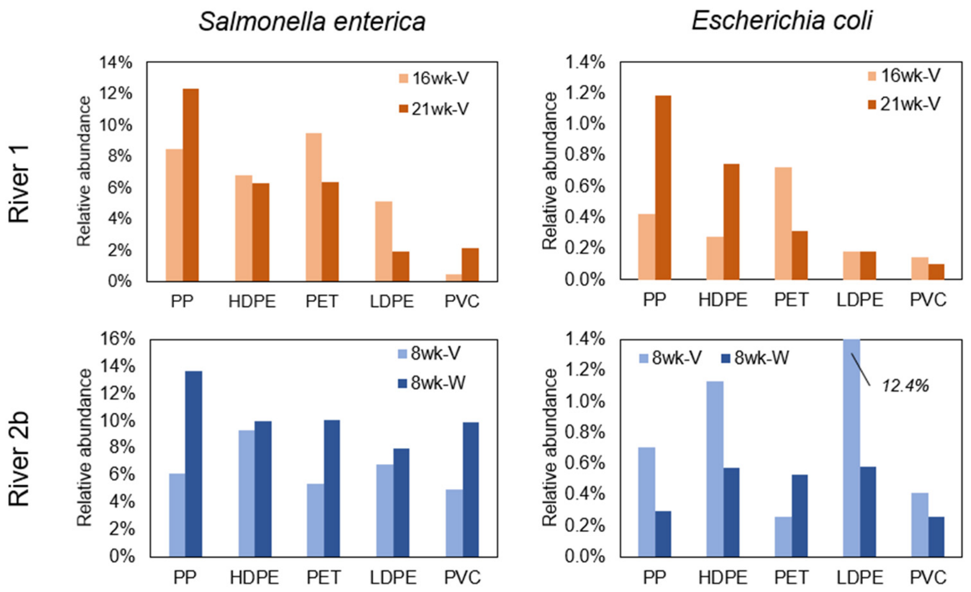

Figure S23. Relative abundance of *Salmonella enterica* and *Escherichia coli* in biofilm associated with Otonabee River and Grand River (downstream). “8wk”, “16wk”, or “21wk” indicate exposure time in weeks, “V” = virgin, “W” = weathered.

References

34. Brandon, J.; Goldstein, M.; Ohman, M.D. Long-Term Aging and Degradation of Microplastic Particles: Comparing in Situ Oceanic and Experimental Weathering Patterns. *Mar. Pollut. Bull.* **2016**, *110*, 299–308, doi:10.1016/j.marpolbul.2016.06.048.

36. Andrade, J.; Fernández-González, V.; López-Mahía, P.; Muniategui, S. A Low-Cost System to Simulate Environmental Microplastic Weathering. *Mar. Pollut. Bull.* **2019**, *149*, 110663, doi:10.1016/j.marpolbul.2019.110663.

46. Fernández-González, V.; Andrade-Garda, J.M.; López-Mahía, P.; Muniategui-Lorenzo, S. Impact of Weathering on the Chemical Identification of Microplastics from Usual Packaging Polymers in the Marine Environment. *Anal. Chim. Acta* **2021**, *1142*, 179–188, doi:10.1016/j.aca.2020.11.002.

67. Gijsman, P.; Meijers, G.; Vitarelli, G. Comparison of the UV-Degradation Chemistry of Polypropylene, Polyethylene, Polyamide 6 and Polybutylene Terephthalate. *Polym. Degrad. Stab.* **1999**, *65*, 433–441, doi:10.1016/S0141-3910(99)00033-6.

68. NOAA - GLERL Great Lakes Coastal Forecasting System (GLCFS) 2020.

69. Gardette, M.; Perthue, A.; Gardette, J.-L.; Janecska, T.; Földes, E.; Pukánszky, B.; Therias, S. Photo- and Thermal-Oxidation of Polyethylene: Comparison of Mechanisms and Influence of Unsaturation Content. *Polym. Degrad. Stab.* **2013**, *98*, 2383–2390, doi:10.1016/j.polymdegradstab.2013.07.017.

70. Rouillon, C.; Bussiere, P.-O.; Desnoux, E.; Collin, S.; Vial, C.; Therias, S.; Gardette, J.-L. Is Carbonyl Index a Quantitative Probe to Monitor Polypropylene Photodegradation? *Polym. Degrad. Stab.* **2016**, *128*, 200–208, doi:10.1016/j.polymdegradstab.2015.12.011.

71. Auta, H.S.; Emenike, C.U.; Fauziah, S.H. Distribution and Importance of Microplastics in the Marine Environment: A Review of the Sources, Fate, Effects, and Potential Solutions. *Environ. Int.* **2017**, *102*, 165–176, doi:10.1016/j.envint.2017.02.013.

72. Atamas’, S.N.; Bukshpun, L.M.; Koptev, Y.V.; Latush, E.L.; Sém, M.F. Stimulated Emission of the 535 Nm Thallium Line as a Result of Quasiresonant Optical Pumping of a Tl–He Mixture by Radiation from a Recombination He–Ca Laser. *Sov. J. Quantum Electron.* **1984**, *14*, 161–162, doi:10.1070/QE1984v014n02ABEH004672.

73. Michael, P. A Conversion Guide: Solar Irradiance and Lux Illuminance 2019.

74. Gewert, B.; Plassmann, M.; Sandblom, O.; MacLeod, M. Identification of Chain Scission Products Released to Water by Plastic Exposed to Ultraviolet Light. *Environ. Sci. Technol. Lett.* **2018**, *5*, 272–276, doi:10.1021/acs.estlett.8b00119.
75. Aslanzadeh, S.; Haghighat Kish, M. Photo-Oxidation of Polypropylene Fibers Exposed to Short Wavelength UV Radiations. *Fibers Polym.* **2010**, *11*, 710–718, doi:10.1007/s12221-010-0710-8.
76. Yang, X.; Jiang, X.; Hu, J.; Wang, F.; Hu, C. Relationship between Physical and Mechanical Properties of Accelerated Weathering and Outdoor Weathering of PVC-Coated Membrane Material under Tensile Stress. *J. Ind. Text.* **2017**, *47*, 197–210, doi:10.1177/1528083716639062.
77. Ministry of the Environment, Conservation and Parks Drinking Water Surveillance Program 2018 - 2020 2020.
78. Natural Resources Canada Photovoltaic Potential and Solar Resource Maps of Canada 2020.
